# Supplementary material for: Content-rich biological network constructed by mining PubMed abstracts
Source: BMC Bioinformatics. 2004 Oct 8;5:147. doi: 10.1186/1471-2105-5-147 (PMC528731; doi:10.1186/1471-2105-5-147)
Supplement: Additional File 2 — The original results of the above study (non-essential files are deleted to keep the file size under the limit set by BMC bioinformatics). [file 1471-2105-5-147-S2.bz2 › chilibotAdditionalFile2/dip10/357ID9062193E4999/html/IL1R1_IL1B.html]

 


 **IL1R1** and **IL1B** 
  
Found 453 abstracts in PubMed, retrieved 10.  
 

 What does Google say? 
 PDF only 
| .edu only 

---

**Interactive relationship** (e.g. stimulation, inhibition, etc)

**Non-interactive relationship** (e.g. studied together, co-existance, homology, etc.)

- DESIGN AND METHODS The associations of a variable number tandem repeat 86 bp polymorphism in intron 2 of interleukin 1 receptor  [ **IL1R1** ]  antagonist IL1 RA and of the 511 C T polymorphism of IL 1b  [ **IL1B** ]  with the risk of CAD were studied.  Ref: 12551827 Haematologica, 2003
- Expression of interleukin 1 beta  [ **IL1B** ]  and interleukin 1 receptor  [ **IL1R1** ]  antagonist in oxLDL treated human aortic smooth muscle cells and in the neointima of cholesterol fed endothelia denuded rabbits.  Ref: 12577317 J Cell Biochem, 2003
